# Respiratory evolution in archosaurs

**DOI:** 10.1098/rstb.2019.0140

**Published:** 2020-01-13

**Authors:** Robert J. Brocklehurst, Emma R. Schachner, Jonathan R. Codd, William I. Sellers

**Affiliations:** 1 School of Earth and Environmental Sciences, University of Manchester, Manchester, UK; 2 Department of Cell Biology and Anatomy, School of Medicine, Louisiana State University Health Sciences Center, New Orleans, LA 70112, USA; 3 Faculty of Biology, Medicine and Health, University of Manchester, Manchester M13 9PT, UK

**Keywords:** lung morphology, breathing, biomechanics, respiratory system, Archosauria

## Abstract

The Archosauria are a highly successful group of vertebrates, and their evolution is marked by the appearance of diverse respiratory and metabolic strategies. This review examines respiratory function in living and fossil archosaurs, focusing on the anatomy and biomechanics of the respiratory system, and their physiological consequences. The first archosaurs shared a heterogeneously partitioned parabronchial lung with unidirectional air flow; from this common ancestral lung morphology, we trace the diverging respiratory designs of bird- and crocodilian-line archosaurs. We review the latest evidence of osteological correlates for lung structure and the presence and distribution of accessory air sacs, with a focus on the evolution of the avian lung-air sac system and the functional separation of gas exchange and ventilation. In addition, we discuss the evolution of ventilation mechanics across archosaurs, citing new biomechanical data from extant taxa and how this informs our reconstructions of fossils. This improved understanding of respiratory form and function should help to reconstruct key physiological parameters in fossil taxa. We highlight key events in archosaur evolution where respiratory physiology likely played a major role, such as their radiation at a time of relative hypoxia following the Permo-Triassic mass extinction, and their evolution of elevated metabolic rates.

This article is part of the theme issue ‘Vertebrate palaeophysiology’.

## 1. Introduction

**Figure 1.**
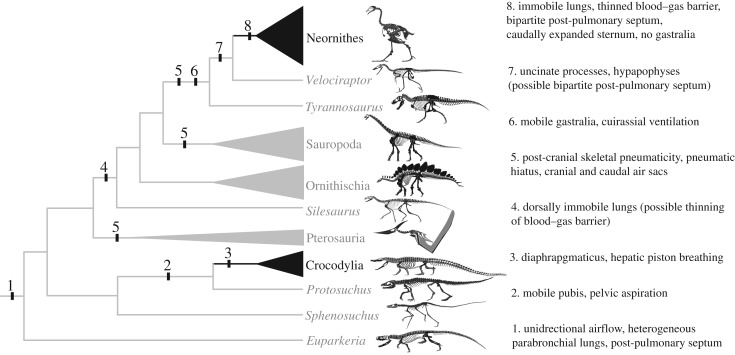
Cladogram of Archosauromorpha, illustrating evolutionary relationships, with major innovations in the evolution of the respiratory system mapped on. Extant taxa are in black, extinct taxa in grey. Skeletal illustrations redrawn from Wilberg *et al*. [2] (*Sphenosuchus* and *Protosuchus*) and Sookias & Butler [3] (*Euparkeria*); all others redrawn from works by Scott Hartman (http://www.skeletaldrawing.com/).

The Archosauria (ruling reptiles) are a highly successful group of tetrapod vertebrates, represented today by birds and crocodylians. Crown-group archosaurs originated over 250 Ma, just prior to the Permo-Triassic Mass Extinction [1]. They then radiated extensively in the Triassic Period, which was marked by the origin of several major clades, including pterosaurs, the first vertebrate lineage to achieve powered flight, and dinosaurs, which came to dominate terrestrial ecosystems for the remainder of the Mesozoic (figure 1). In addition to their evolutionary and ecological success, archosaurs are also marked by the appearance of diverse respiratory and metabolic strategies, providing an excellent opportunity to study the functional evolution of the respiratory system [4] (figure 1).

Birds are the most diverse group of living tetrapods, with over 10 000 documented species; they occupy a wide range of niches and have a global distribution [5]. Additionally, they are one of two vertebrate groups known to have evolved endothermy and one of three to have evolved powered flight [6]. Crcodilians, the living sister-taxon of birds, are much less speciose; all fill similar niches as semi-aquatic, sit-and-wait predators and, like most reptiles, have an ectothermic metabolism [7]. The two clades of extant archosaurs represent the two end-members of the respiratory and metabolic spectrum—ectothermy versus endothermy—with very different activity levels and oxygen demands, and highly divergent respiratory systems and ventilatory mechanisms.

The avian respiratory system is often described as the most efficient of any animal in terms of oxygen extraction [8], and it needs to be in order to cope with the oxygen demands that birds face. Flight is an efficient form of locomotion measured per unit distance travelled, but is expensive per unit time [9] and although locomotion places the greatest demand on the respiratory system in all active vertebrates, this is especially true in flying taxa [10]. Endothermy also greatly increases the amount of oxygen and food an organism must consume [11]. By comparison, crocodilians have relatively low oxygen requirements, but nevertheless, their respiratory system has several unique features, which is curious given their low basal metabolic requirements [12,13]. They are also capable of sustained locomotion, and do not suffer the same locomotor–ventilation constraints as other ectotherms [14].

The metabolic status and levels of activity that could be sustained by fossil archosaurs, particularly non-avian dinosaurs, have long been a matter of considerable debate among researchers, owing in large part to the differences between birds and crocodilians. Almost every possible mode of life has been proposed for non-avian dinosaurs, from full endothermy [15], to reptilian ectothermy [16], to some intermediate condition [17]. Given the intimate association between thermal physiology, respiration and locomotion—as oxygen availability sets a fundamental upper limit on the physiological potential of an organism—a proper understanding of the evolution of the archosaurian respiratory system is critical to our understanding of archosaur physiology. This review will examine the anatomy and mechanics of breathing in the respiratory systems of living and fossil archosaurs, and their physiological implications. The focus will be on bird-line archosaurs as these have been the most well studied, but where possible we will highlight crocodilian-line archosaurs.

## 2. Anatomy of the archosaur respiratory system

### (a) The crocodilian lung

The crocodilian lung is thought to be a better representation of the ancestral lung morphology of archosaurs. This assertion is supported by developmental and anatomical studies which have identified the homologies between the crocodilian and avian respiratory systems [18,19], and where the avian respiratory system seems to be an elaboration on a more basic, crocodilian-like pattern. For example, the branching pattern of the pulmonary bronchi in an adult crocodilian resembles that seen in the embryonic bird lung [20]. The internal airways in the crocodilian lung consist of a single primary bronchus (or air chamber) that gives off multiple secondary bronchi which are all inter-connected by a network of smaller parabronchi, where gas exchange occurs [18,19].

In crocodilians, a post-pulmonary septum (PPS) encloses the lungs in a separate pulmonary cavity away from the abdominal viscera, and acts to support the lung during ventilation [21,22]. Crocodilians have a heterogeneous distribution of gas exchange tissue within the respiratory system, with the densest concentrations in the mediodorsal region of the lung [23]. The more saccular lateral and ventral regions likely play a greater role in ventilation. The heterogeneous lungs of crocodilians should require some attachment to the body wall, preventing the collapse of the more densely partitioned gas exchange tissues in the lung's dorsal region [20,24]. However, there is disagreement over the degree of attachment; Perry [20] described the lungs of Nile crocodiles as fused with the parietal pleura, but Farmer [25, p. 965] described a pleural space surrounding the lungs ‘where the visceral and parietal pleura are readily separated’ in multiple crocodilian species.

Airflow in the secondary bronchi and parabronchi of crocodilians is unidirectional [19,26], a feature once thought to be unique to birds. Unidirectional airflow through the lungs is maintained by aerodynamic valving and is a result of airway geometry in crocodilians [19,26]. The functional implications of unidirectional flow have been reviewed elsewhere [27], and include facilitating economic lung gas mixing and lung gas washout. Unidirectional flow may occur during normal ventilation or through the mechanical coupling of the heartbeat to the lung's ventral cardiac lobe, which overlies the pericardium [28].

Based on features crocodilians share with birds (see below), we can reconstruct the common ancestor of archosaurs as possessing parabronchial lungs with heterogeneously partitioned gas exchange parenchyma, unidirectional airflow through the secondary bronchi and parabronchi and a PPS. Out-group comparisons show that heterogeneously partitioned lungs and a PPS are present in both archosaurs and turtles [29], and unidirectional flow is a basal diapsid character present in archosaurs, turtles and lepidosaurs [27]. Certain lepidosaur lineages have also independently evolved a PPS and high lung heterogeneity, e.g. varanid lizards (monitor lizards) [30–32], and so these animals may represent good extant analogues for lung function in the very earliest archosaurs.

### (b) Avian lungs and air sacs

Bird lungs have several modifications to the ancestral lung morphology. For example, patterns of pulmonary airflow are broadly similar, but birds also combine unidirectional airflow with a cross-current blood–gas exchange pattern [33] which facilitates a reversed blood gas differential, so that pulmonary venous blood can have a higher oxygen and lower carbon dioxide tension than exhaled air. Although the anatomy of the crocodilian lung is consistent with cross-current gas exchange [20,23], there is no experimental data that definitively demonstrate this [25].

**Figure 2.**
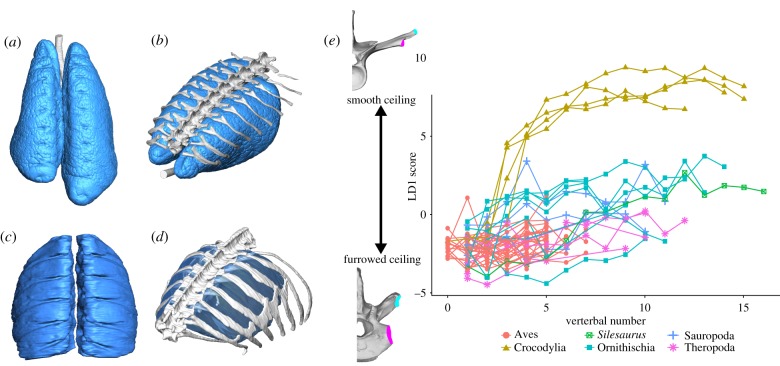
Anatomy of the lung and thorax of extant and extinct archosaurs. Microcomputed tomography (microCT) models of the lungs and ribcage of a hatchling American alligator (*Alligator mississippiensis*) (*a,b*) and an adult African grey parrot (*Psittacus erithacus*) (*c,d*). Dorsal view of the lungs (*a,c*) and in association with the vertebral column and dorsal ribs in left anterolateral view (*b,d*). Plot of vertebral number versus linear discriminant score, separating vertebrae that produce a smooth versus furrowed thoracic ceiling for different archosaur taxa (*e*). Mid-trunk vertebrae for American crocodile (*Crocodylus americanus*) and ostrich (*Struthio camelus*) represent extreme linear discriminant scores. Parapophysis in pink, diapophysis in blue. Modified from Brocklehurst *et al*. [36]. (Online version in colour.)

The major difference in the respiratory system of birds when compared with crocodilians is the complete functional separation of ventilation and gas exchange [8,34]. The lungs of birds are immobile, changing very little in volume throughout the respiratory cycle (less than 1% volumetric change in Pekin ducks [35]), and are entirely devoted to gas exchange. The lung is adhered to the costal wall laterally and vertebral bodies medially [34] (figure 2), and the proximal sections of the ribs incise the dorsal surfaces of the lung, so that approximately 20–33% of the lung tissue lies between successive thoracic ribs [34,37]. The anatomical arrangement of the lungs provides a great deal of structural support, which has permitted greater subdivision of the gas exchange parenchyma into even smaller terminal units for gas exchange, known as air capillaries, and thinning of the blood–gas barrier. Ventilation—movement of air through the lungs—is achieved through the bellows-like action of the air sacs, which are highly compliant and nearly avascular, and do not take part in gas exchange [34].

Tracing the evolution of the avian lung-air sac system in extinct archosaurs requires osteological correlates of the respiratory system or direct evidence of fossilized soft tissues. The air sac system of extant birds is associated with pneumatization of the post-cranial skeleton, namely the invasion of the bones by diverticula (out-growths of the respiratory system). Specific regions of postcranial skeletal pneumaticity (PSP) are associated with the presence of specific air sacs [38,39]. The presence and distribution of pneumatic foramina in the fossilized bones of theropods, sauropods and pterosaurs has been used to reconstruct avian-style air sacs in these taxa and to infer the presence of a bird-like lung [40–43].

However, beyond indicating the presence of air sacs, PSP itself serves no known respiratory function [44] and its extent beyond a basic ‘common pattern’, where only portions of the cervicothoracic vertebral column are pneumatized, is best correlated with body mass or with locomotor behaviours in birds (e.g. it is reduced in diving birds and greater in soaring birds) [39]. In theropod dinosaurs, the extent of PSP increases with body mass but the threshold at which it increases is lower in maniraptorans, which may be an adaptation to high metabolic rates by replacing energetically expensive bone with air [43]. Much of the literature on patterns of PSP and the spatial arrangement of air sacs focuses on whether air sacs cranial and caudal to the lungs indicate the presence of unidirectional flow [40,42]. However, this overlooked previous experimental work which showed that unidirectional airflow is the product of aerodynamic valving and airway geometry [45–47] and that occlusion of the thoracic and abdominal air sacs did not affect the patterns of pulmonary airflow in birds [48,49]. Unidirectional flow is now also known from multiple non-avian sauropsids that lack extrapulmonary air sacs, e.g. crocodilians [19,25,26], *Varanus exanthematicus* [31] and *Iguana iguana* [50].

The evolution of the immobile avian lung can be traced using the anatomy of the costovertebral joint. Both birds and crocodilians share bicapitate ribs, with two articulations to the vertebral column at the costovertebral joint—the rib capitulum with the vertebral parapophysis and the rib tuberculum with the vertebral diapophysis [51,52]. In crocodilians, the anterior-most thoracic vertebrae have the parapophyses on the vertebral centra, and the diapophyses on the ends of the transverse processes. The ribs that articulate with these vertebrae are strongly forked. From the third thoracic vertebra, the parapophysis migrates onto the transverse process towards the diapophysis; the tuberculum becomes reduced and the ribs get less forked [13] (figure 2). Eventually, the two articulations fuse and disappear [13] (figure 2). This creates a smooth thoracic ceiling and the crocodilian lung has a smooth dorsal surface (figure 2). In birds, the parapophysis is located on the centrum for the entire dorsal series, and the ribs are all strongly forked. This creates a furrowed thoracic ceiling, where the dorsal surface of the lungs is deeply incised by the rib capitulate, immobilizing the lung's dorsal surface (figure 2).

Qualitative and quantitative comparisons of the ribs and vertebrae in both non-avian dinosaurs and the non-dinosaurian dinosauriform *Silesaurus* found that they possessed a furrowed thoracic ceiling, with forked ribs which would have incised the lung's dorsal surface and rendered dorsal components of the lung immobile [36,53] (figure 2), similar to modern birds. The lungs in these taxa would have had a great deal of structural support, and so would have fulfilled the functional prerequisites for the increased subdivision of the parabronchi and thinning of the blood–gas barrier seen in the lungs of extant birds [8]. Further investigations into the presence of these osteological correlates in other fossil taxa (e.g. stem archosaurs, fossil pseudosuchians) are still needed, however, to complete the picture of lung evolution.

In birds, the PPS is bipartite, as it is invaded by the air sacs during development and split into two [54]. The horizontal septum borders the lungs ventrally, isolating them within the pleural cavity, whereas the oblique septum divides the remaining space into the sub-pulmonary (contains the majority of the air sacs) and abdominal cavities (contains the viscera and abdominal air sacs) [30,34]. The median fibres of the horizontal and oblique septa attach to the hypapophyses, ventral processes on the cervico-dorsal vertebrae [34]. Based on the presence of well-developed hypapophyses in maniraptoran theropods, it has been hypothesized that these taxa had a bipartite PPS which would have provided additional support ventrally for a rigid immobile lung, and completed the division of the respiratory system into discrete ventilator and exchanger [30]. However, the hypapophyses are also present in crocodilians, and in both birds and crocodilians serve as an attachment for the longus colli muscles [55,56], so this remains somewhat speculative.

Direct evidence for lung structure in extinct taxa can only come from preserved soft tissues. Fossilized lung tissues are known from the basal ornithuromorph bird *Archaeorhynchus*, which, despite being approximately 125 Ma, shows very close similarities with the lungs of extant birds [57]. The presumptive lung tissue shows impressions made by the ribs, similar to the costal sulci in the lungs of living birds [57]; this intimate association with the ribs and direct attachment of the lung to the body wall would have rendered it immobile, facilitating increased subdivision of the gas-exchanging parenchyma and thinning of the delicate blood–gas barrier. Scanning electron microscopy revealed tissue structure of the parabronchial lumen and surrounding regions—which closely resembles the air capillaries and terminal gas exchange units of an ostrich lung [57]. The general structural design of the avian lung, therefore, has been conserved for some time.

## 3. Evolution of ventilation mechanics in archosaurs

### (a) Costal aspiration in living and fossil archosaurs

Both birds and crocodilians use costal aspiration—changes in the volume of the ribcage driven by rib motions and the axial musculature—to ventilate their lungs [58]. In both groups, the ribs are bicapitate and form two distinct articulations with the ribcage: the capitulum-parapophysis and tuberculum-diapophysis [53,59]. Theoretically, the bi-condylar costovertebral joint should behave like a hinge, with the parapophysis and diapophysis forming an anatomical axis of rotation that constrains the motion of the vertebral ribs. However experimental evidence from XROMM (X-ray reconstruction of moving morphology) of the ribcage in American alligators (*Alligator mississippiensis*) demonstrated that costovertebral joint anatomy predicted overall patterns of motion across the ribcage, but there were significant deviations and generally, ribs *in vivo* rotate about all three body axes more equally than predicted [60]. By contrast, similar studies in birds, wild turkeys (*Meleagris gallopavo*), found that a hinge-like model of joint motion was well supported [61].

Given the important role costal aspiration plays in ventilation in both birds and crocodilians [13,60,62,63], the reconstruction of rib motion is key to reconstructing ventilation as a whole. As the dorsal vertebrae and vertebral ribs are ossified in all archosaurs, they are frequently preserved in the fossil record and with them the anatomy of the costovertebral joint which can inform predictions of rib motion [63]. Although the ventral elements of the ribcage—the sternum and sternal ribs—are only ossified and preserved in certain taxa (e.g. maniraptorans and pterosaurs [64,65]), they would have been connected to the vertebral ribs in life, and so the anatomy of the costovertebral joint can still be a useful correlate of rib mobility and breathing mechanics.

In non-avian dinosaurs, the morphology and orientation of the costovertebral joint has been shown to be very similar to extant birds, both qualitatively [53,59] and quantitatively [36]. Additionally, some dinosaur taxa preserve evidence for the soft tissue anatomy of the costovertebral joint, in the form of ligamental scars on the vertebrae and rib heads [66]. Unlike in crocodilians [60], predictions of rib motion based on costovertebral joint anatomy matched well with the actual *in vivo* motion of the ribs in birds [61]. Based on the greater predictive power of anatomy in birds, and the greater anatomical similarity of non-avian dinosaurs to birds, then we should be able to accurately reconstruct rib motion in dinosaurs.

### (b) Evolution of avian ventilation mechanics

**Figure 3.**
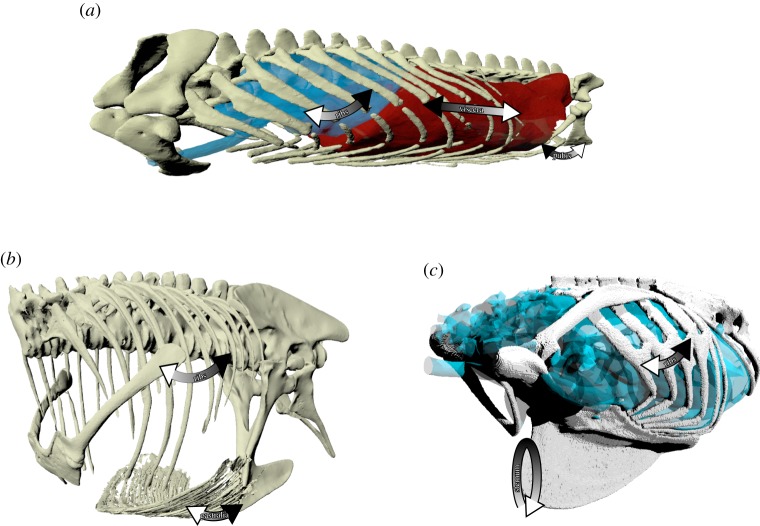
Respiratory mechanics of archosaurs. Oblique views of an American alligator (*Alligator mississippiensis*) showing the trunk skeleton, lungs and viscera (*a*); the ribcage and gastralia of *Tyrannosaurus rex* (TCM 2001.90.1) (*b*); and the skeleton and lung-air sac system of a parrot (*Psittacus erithracus*) (*c*). Arrows indicate skeletal and visceral movement during ventilation: white is inspiration; black is expiration. (Online version in colour.)

In addition to the mobile ribcage in the thorax, the dinosaurian ancestors of modern birds possessed mobile gastralia in the abdominal region [67] (figure 3). It has been hypothesized that contraction of the pelvic and hypaxial musculature could have narrowed and widened the gastral basket to ventilate the caudal air sacs or caudal regions of the lung [64,68]; lateral rotation of the gastralia in *Allosaurus* increased abdominal cavity volume by 14% [68]. Basal birds retain gastralia, e.g. *Archaeopteryx*, but they are lost in more advanced ornithurine birds [67]. Loss of gastralia coincides with the caudal expansion of the sternum, which has replaced the gastralia in modern birds, both functionally and topographically [67,69] (figure 3).

Most species of birds possess uncinate processes (UPs), accessory breathing structures that project off the vertebral ribs [70] (figure 3), increasing the mechanical advantage of the inspiratory appendicocostalis and expiratory external oblique muscles, and their morphology is correlated with locomotor mode and metabolic rate [71,72]. Increasing the angle of the ribs to the vertebral column will increase the mechanical advantage of the appendicocostalis (decreasing the benefits of UPs), and increasing the length of the sternum and pectoralis will increase the force necessary for sternal depression during inspiration [71]. Diving birds have ribs at a low angle to the spine (to improve streamlining during diving), a long sternum and large pectoralis, so they have long UPs [71]. Load-carrying experiments showed that diving tufted ducks (*Aythya fuligula*) experienced less of an increase in metabolic rate when mass was added to the sternum compared with geese (*Branta leucopsis*), possibly owing to their long UPs, and the improved leverage they provide [73].

Cartilaginous UPs are present in extant crocodilians and serve as attachments for the expiratory iliocostalis muscle [74]. UPs are also present in the fossil record of stem birds and non-avian theropods, so their function as levers for the respiratory muscles predates the origin of flight [64]. The UPs of non-avian theropods are relatively long compared with the size of the ribcage and the proportions are more similar to living flying or diving birds than running species [64]. The processes in these taxa may have articulated with the ribs via a flexible cartilaginous joint, which would have reduced the efficiency of force transmission [75], necessitating longer UPs. The presence of UPs may have helped keep cost of ventilation down in early birds even as the sternum became specialized for flight.

### (c) Hepatic piston ventilation in crocodilians

Crocodilians possess several unique components to their respiratory system that contribute to ventilation [13]. The most important is the hepatic piston diaphragm driven by the action of the diaphragmaticus muscle, which pulls the liver caudally, increasing pleural-cavity volume and driving inspiration [12,13,76] (figure 3). In juvenile crocodilians, the diaphragmaticus serves an accessory role in lung ventilation, supplementing the intercostal musculature during times of increased breathing resistance or energetic demand, e.g. during digestion or exercise [76,77]. Transection of the diaphragmaticus had no significant effect on breathing rate at rest, demonstrating that costal breathing alone is capable of supporting resting metabolism [76,77]. Older, larger crocodilians, by contrast, may rely almost completely on the hepatic piston pump [78]. These differences associated with age may be related to decreased body wall compliance as crocodilians grow and the body wall becomes more muscular and keratinized [79]. Further testing is still needed to determine regional differences in body wall compliance, and to distinguish between expansion of the abdomen (hepatic piston) and thorax (costal aspiration).

The origins of the crocodilian hepatic piston remain a mystery as it has no confirmed osteological correlates. The evolution of the pubic mobility is well documented in the fossil record [80] (figure 3), and was thought to stop the abdominal viscera getting squeezed by caudal displacement of the liver, preventing dangerous increases in abdominal pressure and reductions in venous return from the hindlimbs [12]. However, experimental immobilization of the pelvis in alligators did not significantly increase intra-abdominal pressure or decrease venous return [81], and there is no strict correlation between pubic rotation and visceral displacement caused by the diaphragmaticus [13]. Therefore, pelvic mobility cannot be used as an osteological correlate for the hepatic piston [80].

The smooth thoracic ceiling described above (§2b) would facilitate hepatic piston breathing, by providing a smooth surface along which the lung tissues can slide as it inflates and deflates in the cranio-caudal direction [36,53] (figure 2). Although the crocodilian lung is attached to the body wall craniodorsally, the caudal regions of the lung are flexible, and radiographic data clearly show the caudal border of the crocodilian lung moving craniocaudally during ventilation [13,60]. The precise interactions between the lung surface and the vertebrae (i.e. between the visceral and parietal pleura) in this part of the trunk during breathing, however, remain currently unknown.

There is also the case for whether the hepatic piston evolved in terrestrial or aquatic fossil crocodilians. Extant crocodilians use the hepatic piston to supplement costal aspiration during running exercise, helping circumvent Carrier's constraint [14]. However, they also use the hepatic piston to control the position of the centre of mass and buoyancy during aquatic locomotion [82]. Given the lack of reliable osteological correlates for the diaphragmaticus or the hepatic piston, and the general lack of data on stem crocodilians, it is very difficult to say when these features evolved and for what purpose.

### (d) The problem with pterosaurs

Of all fossil archosaurs, pterosaurs are some of the most problematic for reconstructing ventilation. Pterosaurs have a bipartite ribcage, and the sternal ribs increase in length caudally, similar to birds [65]. In certain taxa, the vertebral ribs fuse to the thoracic vertebrae forming a synthorax [83], which would seem to preclude costal aspiration. The presence of elongate sternal ribs suggests that the sternum was still capable of significant displacement [65,84], but this may have been constrained by articulations between the sternum and pectoral girdle [83]. The sternal ribs in pterosaurs also possess sternacostapophyses [65], similar to those present in *Sphenodon* [64], which may have acted as levers to increase the moment arm for the intercostal muscles (analogous to avian UPs). PSP has been used to reconstruct bird-like air sacs [65], but qualitative assessments of costovertebral joint anatomy suggest a more crocodilian-like compliant lung [83].

The presence of a visceral pump in pterosaurs [83] seems unlikely. In birds, inspiration and expiration occur primarily by the dorsoventral rocking of the sternum [62]; breathing in this way does not affect pitch or roll during flight as most changes in the position of the centre of mass occur in the dorsoventral plane, promoting stability [44]. By contrast, during hepatic piston breathing, the centre of mass in crocodilians shifts substantially in the craniocaudal direction [82]. If pterosaurs breathed using a similar visceral pump mechanism, they would likely be highly unstable during flight. This hypothesis remains to be tested, however, and does not rule out an alternative extra-costal mode of ventilation which expanded the lungs and air sacs in the dorsoventral plane to avoid affecting stability.

## 4. Oxygen physiology and archosaur evolution

### (a) Hypoxia tolerance and the Permo-Triassic mass extinction

The Permo-Triassic (P-Tr) mass extinction was the most devastating mass extinction in the history of life on Earth, and resulted in major faunal turnover in both terrestrial and marine environments [85]. On land, the synapsids had dominated in the Permian and were replaced by archosauromorph diapsids. This followed a period of recovery during the aftermath of the extinction in the Early Triassic, resulting in an extensive radiation in the Middle and Late Triassic [86,87]. Many hypotheses have been put forward to explain this faunal turnover, and why archosauromorphs were more able to thrive in the post-extinction world of the Triassic than were the surviving lineages of synapsids [87].

One key environmental difference between the Permian and the Triassic was the levels of atmospheric oxygen. Geoclimate models show that the Permian was hyperoxic compared with the present day and show a decrease in atmospheric oxygen across the P-Tr boundary [88,89]. However, different models disagree on how sharp this decrease was and whether levels in the Triassic were lower than [88] or equal to [89] those in the present day. Levels of atmospheric oxygen are important as, when comparing living animals, extant synapsids with a bronchoalveolar lung, i.e. mammals, cannot tolerate hypoxic conditions as well as modern birds can with a parabronchial lung [90]. The most important reason for this difference is the significantly thinner blood–gas barrier in birds [28]. It has been hypothesized that the parabronchial lungs of early dinosaurs and other archosaurs provided them with an advantage in the relatively hypoxic conditions of the Triassic [28,88,91], allowing them to better survive and recover in the wake in the extinction event.

However, this hypoxia scenario is not well supported by the fossil record. Many faunas were still synapsid dominated in the immediate aftermath of the P-Tr extinction [92], and some Triassic synapsids grew to large sizes similar to their archosaurian contemporaries [93]. Archosaurs did not become the main faunal components in terrestrial ecosystems until the Late Triassic [94], 30 Ma after the P-Tr extinction, and the most common archosaurs until this point were crocodile-line curotarsans [94] for which we have many fewer data on possible lung morphology and function. For this evolutionary scenario to be considered plausible, evidence is needed from: diversity studies to show the pattern of faunal turnover, taking into account biases in the fossil record; abundance surveys, to assess which taxa were dominant numerically and ecologically; a well-dated phylogeny with acquisition of key characters time-matched to diversification events or shifts in environmental conditions; and a biological mechanism. Comparing extant archosaurs at least provides us with a plausible biological mechanism in terms of hypoxia tolerance [90], but this assumes that fossil synapsids had lungs that behaved in a similar way to modern mammals. The links between oxygen levels and the archosaur radiation are intriguing, but require much more rigorous testing following the roadmap outlined above.

Whether tolerance of hypoxia shaped the evolution of the avian lung—with its thin blood–gas barrier and highly subdivided parabronchi—in later bird-line archosaurs from a more crocodilian-like ancestral state could be tested using extant animals. It has been shown that American alligators grown under hypoxic conditions post-hatching have accelerated lung growth and larger lungs relative to body size [95]. However, whether changes occurred in the lung structure of these animals—for example, increased parabronchial subdivision—was not investigated. Andean geese, which live under hypoxia at high altitude, show increased mass-specific respiratory surface area, subdivision of the lung parenchyma and overall oxygen diffusing capacity compared with their lowland relatives [96]. However, birds increase cardiac output in response to hypoxia [97], and so species that live in severely hypoxic environments actually show a thickened blood–gas barrier to prevent structural failure [96].

### (b) Endothermy in archosaurs

There is now a great deal of evidence that fossil archosaurs had metabolic rates elevated above those of modern ectotherms, including extant crocodilians. Multiple lines of evidence support this, such as studies of bone histology and growth rates [17,98], oxygen isotopes [99], blood-vessel foramina [100] and cell and genome size [101,102]. However, there has been little quantitative modelling of the respiratory system to evaluate how these animals might have met the oxygen demands associated with an endothermic metabolism, or how they might have dealt with the other physiological consequences of endothermy, such as heat and water conservation [103].

Early attempts to theoretically model the respiratory system of dinosaurs used equations for oxygen uptake in the mammalian lung [104], and respiratory parameters from extant monitor lizards to estimate maximum oxygen uptake and delivery (VO_2_ max), and compare this with modern endotherms and ectotherms [105]. Slight alterations to physiological parameters of the model based on ranges of values seen in extant ectotherms were capable of producing significant increases in VO_2_ max, and so early endotherms would not have been constrained for possessing non-avian lungs [105]. Other models have since been developed that better approximate the avian respiratory system e.g. by accounting for cross-current gas exchange [106], but given the uncertainty on the gas exchange mechanism in crocodilians, it is unclear which model is most appropriate for fossil archosaurs.

## 5. Conclusion

Archosaurs show a unique set of adaptations to their respiratory systems, and it is intriguing to think that these were major keys to their success. A heterogeneous lung with a thin blood–gas barrier and unidirectional airflow likely provided them with greater oxygen extraction and hypoxia tolerance, allowing them to survive and radiate after the Permian–Triassic extinction event. Coupled with efficient ventilation, this allowed them to unlock other physiological innovations such as powered flight and an endothermic lifestyle. Modern techniques, e.g. morphometric analyses and XROMM, have provided quantitative insights into the relationship between skeletal form and lung structure and ventilation mechanics, which can be applied to fossils. Experimental physiology of crocodilians and other non-avian sauropsids has provided more data on the phylogenetic distribution of physiologically important traits, and comparative studies across birds allow us to test hypotheses on ecology and respiratory performance. However, some questions remain unanswered, either because the relevant fossil material has yet to be examined for the necessary osteological correlates (e.g. lung structure in the earliest archosaurs or the origins of the crocodylomorph hepatic piston), or because hypothetical functional scenarios require testing using biomechanical models (e.g. ventilation and flight in pterosaurs). Given the fundamental role of oxygen in metabolism, and the intimate association between respiration, thermal physiology and locomotion, respiratory biology of extinct groups, and particularly archosaurs, is a valuable research area in vertebrate palaeophysiology.

## Data accessibility

This article has no additional data.
